# Process Mining for Individualized Behavior Modeling Using Wireless Tracking in Nursing Homes

**DOI:** 10.3390/s131115434

**Published:** 2013-11-11

**Authors:** Carlos Fernández-Llatas, José-Miguel Benedi, Juan M. García-Gómez, Vicente Traver

**Affiliations:** 1 Instituto Universitario de Investigación de Aplicaciones de las Tecnologías de la Información y de las Comunicaciones Avanzadas (ITACA). Universitat Politècnica de València, Camino de Vera S/N, Valencia 46022, Spain; E-Mails: juanmig@ibime.upv.es (J.M.G.-G.); vtraver@itaca.upv.es (V.T.); 2 Unidad Mixta de Reingeniería de Procesos Sociosanitarios (eRPSS), Instituto de Investigación Sanitaria del Hospital Universitario y Politécnico La Fe, Bulevar Sur S/N, Valencia 46026, Spain; 3 Instituto Tecnológico de Informática (ITI), Universitat Politècnica de València, Camino de Vera S/N, Valencia 46022, Spain; E-Mail: jbenedi@dsic.upv.es

**Keywords:** process mining, individualized behavior modeling, ambient assisted living, ILS processing

## Abstract

The analysis of human behavior patterns is increasingly used for several research fields. The individualized modeling of behavior using classical techniques requires too much time and resources to be effective. A possible solution would be the use of pattern recognition techniques to automatically infer models to allow experts to understand individual behavior. However, traditional pattern recognition algorithms infer models that are not readily understood by human experts. This limits the capacity to benefit from the inferred models. Process mining technologies can infer models as workflows, specifically designed to be understood by experts, enabling them to detect specific behavior patterns in users. In this paper, the eMotiva process mining algorithms are presented. These algorithms filter, infer and visualize workflows. The workflows are inferred from the samples produced by an indoor location system that stores the location of a resident in a nursing home. The visualization tool is able to compare and highlight behavior patterns in order to facilitate expert understanding of human behavior. This tool was tested with nine real users that were monitored for a 25-week period. The results achieved suggest that the behavior of users is continuously evolving and changing and that this change can be measured, allowing for behavioral change detection.

## 1. Introduction

During the last few years, there has been an increasing interest in the study of human behavior outside of classic application fields (medicine, health, etc.). There is evidence in the literature [1,2] demonstrating the importance of the study of human behavior in various fields. For example, early diagnosis of dementia can be made through the detection of changes in social habits [3]. In addition to that, the literature advises that identification of behavior models may also increase adherence to drug treatments and improve healthy habits via individualized motivational techniques [4–6].

Studies available in the literature are performed by creating generalized models. However, the social and physical make-up of human beings requires very adapted models for efficient and effective motivation. It has been demonstrated that individualized motivation techniques are the most suitable for promoting behavior changes [7]. As a result, the creation of an individualized behavior model is needed in order to apply the correct strategies for health promotion to each individual. The design of this kind of model is not a trivial task. Usually, the experts manually model the user's behavior after observing the behavior patterns of the user for a prolonged period of time (months or even years). This methodology has two important disadvantages: firstly, it requires too many human resources and far too long a time, and secondly, the final result does not reflect the current status of the person, due to the influence of time.

Pattern recognition can be a solution to solve that. Pattern recognition algorithms [8] have been successfully used in other research fields as an alternative to the classical deductive manual approach. The pattern recognition paradigm is based on the use of algorithms to identify the inherent model through acquired samples. As a result, it is possible to infer a mathematical model of human behavior by collecting and processing the data of human actions using pattern recognition techniques. Pattern recognition algorithms can help experts in the design of models by transforming the raw data of individual annotated actions into models that explain or classify their behavior.

Although pattern recognition algorithms can provide a good approach to infer behavior models that help experts in the discovery of processes, usually the inferred models for these algorithms are difficult for experts to understand. The classic algorithms (neural networks, hidden Markov models, etc.) use complex mathematical models to represent the inferred knowledge. This makes it difficult to correct the inferred models by using heuristics and to even understand the model itself. This problem is addressed by the use of various techniques, such as IPR (Interactive Pattern Recognition) [9]. An example of that is computer-assisted translation (CAT, [10]). This paradigm was initially presented as a way of increasing the effectiveness of the whole learning process in order to incorporate human correction activities within the learning process itself. In certain research fields, such as machine translation, this paradigm has been successfully proposed to improve the accuracy of learning processes [10]. In [10], an iterative process is presented, in which, in each iteration, a data-driven machine translation engine suggests the completion for a prefix of a target sentence, which a human translator can accept, modify or ignore. According to this paper, the proposed techniques reduce the effort needed to produce a high-quality translation from a given source text by up to 80%, in comparison to the effort needed to simply type the whole translation. This model can also be applied to create algorithms to solve problems in which the final modules need to be understood by experts. In that case, the inference systems of human behavior, making use of the CAT approach, will allow for human comprehension of the results of the algorithms. This approach allows experts to modify them to improve and enrich the models with the incorporation of expert knowledge. The creation of human understandable models is one of the objectives of process mining technology.

Process mining (also known asworkflow mining) [11,12] is a technology that allows workflow inference from event or activity logs. A workflow [13] is a formal representation of a process designed to be automatized. That means that process mining technology can be used to infer graphs understandable by human experts (workflows) using the daily actions collected by ambient intelligence (AmI) environments. This allows the experts to understand the behavior process of the individual and to compare it with previous inferences in order to detect specific behavior changes and patterns.

Classical process mining algorithms, like PALIA (parallel activity-based log inference algorithm) [14], the alpha algorithm [11], heuristic miner [15] or the genetic process mining algorithm [16], have been previously tested in laboratory conditions in previous works [14,17]. These works conclude that PALIA was the best algorithm in accuracy and understandability. The research done in this paper is a continuation of this work, so a comparison among the different process mining algorithms is out of the scope of this paper.

Pattern recognition algorithms require the collection of data in order to infer a behavior model of the user. However, if the collection process is too intrusive, it can influence the behavioral model of the user, inducing undesirable artificial patterns. As a result, in order to make use of pattern recognition algorithms, a continuous and transparent system for collecting individual logs is needed. The infrastructure of information and communication technologies (ICT) currently available permits the creation of intelligent environments that allow the collection of large sets of data from daily user actions. In particular, the ambient intelligence (AmI) [18] concept, which is currently popular in research fields, such as, ambient assisted living (AAL) or smart cities, is a good alternative to the manual collection of information. The AmI paradigm is thought to provide intelligent environments to empower people by collecting continuous information on human activity. The raw information in this kind of system is produced by the sensors that are deployed. This information is the basis of the creation of behavior models of each user by using pattern recognition algorithms. These models have direct application in several fields, like the simulation of specific users to test human adaptation to products being designed [19], the detection of conduct disorders of individual patients in their living environments [20] or the discovery of care protocols for specific illnesses [14].

In this paper, our work is focused on extractable and graphically represented human understandable information from the individual behavior of real users in ambient assisted living environments, while being as little intrusive as possible. This information can be used by human experts, according to IPR principles, to extract individualized behavioral knowledge with these data. Our hypothesis is that physical displacements of users in AAL environments can offer information about the individual behavior of the user, and the comparison of this behavior in time could be a measure of the behavioral change of an individual person. Specifically, we capture all the areas (rooms in a nursing home) visited by the user over time.

In this way, the research presented in this paper is focused on the processing of users' location information in order to support human experts with the discovery of individual behavioral knowledge of real users at risk of dementia. This information was captured using ILS (indoor location system) sensors deployed in a nursing home. In this work, we present a set of algorithms based on process mining techniques that helps professionals infer and compare individualized visual models of human behavior. In this paper, the authors continue the research by using these techniques in real environments and formalizing the workflow comparison algorithms. This research is done under the umbrella of the eMotiva Project. eMotiva is a Spanish Government-funded project whose main objective is the creation of a motivational platform for patients suffering from dementia. In [21], the authors presented a preliminary paper of the project with some process mining technologies tested in lab conditions.

In our experiment, we monitored the location of nine residents of a Spanish nursing home for a 25-week period. By using this data, we expect to show to experts specific situations inferred from habitual movement patterns, such as user location preferences, time spent in specific places, etc. In addition, comparing these behavior models with previously inferred models, it is possible to help human experts discover specific behavior changes of the individual. These behavior changes can be incremental or sudden.

This paper is structured as follows. Firstly, a brief review on location systems and behavior modeling algorithms is given. Secondly, the proposed system and the algorithms selected and implemented are presented in detail. Thirdly, the results of experiments with real users are shown. Finally, the paper ends with a discussion and conclusion section, where the results achieved and future work are discussed and summarized.

## 2. Related Work

In the literature, there are studies that use human tracking information to make new discoveries. In [22], a video camera was used to detect specific movements of humans. More recently, in [23], an array of ultrasound sensors was used to monitor human movement in a room for psychological experiments. The use of video cameras or arrays of sensors is often employed to monitor limited spaces in one or two rooms, due to their high cost, and so, the use of those systems to track residents in all rooms of a nursing home requires a high investment. In addition, the use of video cameras raises a lot of ethical issues, and so, the use of such technologies was discarded.

There are other technologies for tracking users. In the literature, there is work based on outdoor tracking using GPS (global positioning dystem) [24–26]. However, GPS technology does not work in indoor spaces. For our research, a solution based specifically on an indoor location system (ILS) is required. Although ILSs are still being researched [27,28], there are commercial products available that provide ILS, such as the Sphera System [29]. The Sphera ILS System is a stable commercial system that has been installed in big health centers, like Hospital La Fe of Valencia (Spain). This system has a tested battery autonomy of more than one year. In our experiment, we needed a reliable ILS system, with experience in health scenarios and with a very high autonomy, because the changing of batteries can affect the behavior of the residents. For all these reasons, the Sphera System was selected to provide location data in the eMotiva Project.

The use of pattern recognition algorithms to process location events produced by the Sphera System to automatically infer individualized behavior models is an open issue. In the literature, certain paradigms exist that deal with the detection of specific behavior patterns in corpora with a large quantity of events.

One of these approaches is called complex event processing [30]. Complex event processing (also known as CEP) allows us to process events and discover complex patterns existent among multiple streams of event data. In this model, all events are processed in order to find specific patterns pointing to specific behavior. As a result, it is possible to define syntactical patterns that are used by search engines in order to find event sequences that are similar to them and, then, to detect specific behavior in the continuous stream of user actions. However, the models inferred by CEP technologies are not thought to be understood by experts; and it can be very difficult for them to find specific patterns or changes in the behavior models.

Another approach to this framework is the use of plan recognition models [20]. In this framework, the experts begin by manually describing the flow that they want to discover. The algorithms automatically align the behavior corpus with this flow in order to separate the events depending on the state of the defined flow that they represent. For example, if the experts describe the complete flow of a user divided into states (sleeping, having lunch, etc.), the algorithm aligns the described flow with the raw information gathered from AmI sensors in order to detect the sequence of events that refer to each state. In this way, the aligned model allows the detection of specific situations that can be understood by human experts. The main problem with this approach is that the flow of the user must be initially described by experts, and this is not the case for our research, because the flow is unknown.

The previous approaches are focused on the detection of specific situations in the flow of continuous action. However, these algorithms do not allow experts to discover the whole process from scratch. Understanding of the complete models will allow behavior experts to navigate through the whole process and look for individual characteristic user patterns. To achieve this, it is mandatory that the resultant model be formally described in a manner understood by experts. Process mining is a technology that can deal with this [12]. Process mining algorithms identify processes represented as workflows from the data log of actions. Workflows are formal representations specifically made to be understood by experts. In this way, the process mining idea sacrifices the accuracy of the inference algorithms in order to provide human experts with an understandable view of the process. In our case, in AmI environments, all the actions collected can be used by process mining algorithms to describe the whole process of user actions using workflows as the representation language. As a result, this paradigm can be used to infer and represent the whole behavior model of the user [17].

There are several algorithms in the literature able to deal with this paradigm. One of the first algorithms provided by the literature is the alpha algorithm [11]. Alpha is a heuristic algorithm thought to infer complex workflow models from event samples. The alpha algorithm is able to provide Petri nets [31] from the events produced in the logs. However, the complexity of Petri nets makes understanding of the processes inferred by the experts, who are more habituated to simpler workflow languages, like finite automatons, more difficult. Other algorithms with simpler approaches have been presented. The heuristic miner [15] algorithm is an algorithm that infers directed graphs using heuristic techniques based on frequencies of events and sequences. Heuristic miner removes infrequent paths in order to provide a more readable and meaningful view of the processes. The use of heuristic algorithms allows for a more quick and direct way of solving the inference of models. However, the use of heuristics in pattern recognition algorithms can compromise their use in different research fields. In that way, to ensure that the heuristic is able to be used in a specific problem, one needs to ensure that the assumptions taken into account in the algorithm are admissible in each of the fields where the algorithm is used, in order to be sure about the applicability of the algorithm. This is crucial in fields where the real characteristics of the problem are unknown, like in individualized human behavior modeling. However, not only heuristic techniques have been used to infer workflows. Based on the genetic algorithms framework, the genetic process miner algorithm was enunciated [16]. The genetic process miner is able to infer directed graphs in an evolutionary way. However, genetic process mining has some limitations. The inference of directed graphs does not allow for the inference of parallel activities and sequences, due to the features of the final language that is inferred. Another non-heuristic algorithm able to infer more complex patterns, like parallel sequences, is PALIA [14]. PALIA is based on the grammatical inference pattern recognition framework [32]. This algorithm infers workflows described as timed parallel automatons (TPA) [33]. TPA is a formal framework for defining highly expressive workflows, as expressive as safe Petri nets [33], having a regular grammatical complexity [33]. This allows for the definition of more complex workflow patterns, like parallel sequences, described as understandable finite automatons. Those algorithms were tested in previous works, PALIA being the most accurate algorithm [14].

In addition, classic process mining applications in business processes are intended to avoid infrequent events in order to allow experts to extract only the meaningful information, removing the noise from the worflows discovered [12]. However, infrequent information is important in the daily movements of residents. For example, a visit to the hairdresser can be only once a month, and according to the classical view, this fact should be removed. However, this visit might be crucial for detecting a change in behavior of the resident. For example, forgetting the hairdresser visit can be a symptom of depression or social withdrawal. PALIA can be configured for providing a complete TPA with statistical information about the frequency of activities and state changes, but also including infrequent behavior.

In our experiment, we gather location information from individual residents in a nursing home. We assume that the human behavior is continuously evolving. According to that, it is supposed that the model that we can infer will be different depending on the period of time during which we take the sample. This behavioral change of processes, or concept drift, has been treated in the process mining literature. Bose et al. [34] present an algorithm for detecting sudden drifts based on heuristical distances, taking into account the precedence among activities. According to this work, the use of distances can be a good way to detect changes in the evolution of processes. However, this distance measure works in problems in which the precedence among activities is clearly defined, like business processes. However, this might not be acceptable in a model like individualized human behavior, where that precedence is not so clear. In addition, the proposed distance method detects when a change is produced, but it is not able to show the sequence of activities that are causing the change in the process. This information can be very useful for physicians in order to know what changes are occurring in the resident's behavior. In that case, we need a more adequate workflow distance algorithm for our problem that is able to discover the behavioral sequences that changed. These changes should be highlighted in the proper inferred workflow to support experts in the discovery of the causes of the behavior change. In this work, we present a structural distance algorithm based on the ECGI (error correcting grammar inference) formal framework [35], which is intended to detect structural changes in the workflow. This algorithm not only presents a numeric measure of the distance, but also presents the differences in a graphical way that can be understood by experts.

## 3. Sphera Indoor Positioning System

**Figure 1.**
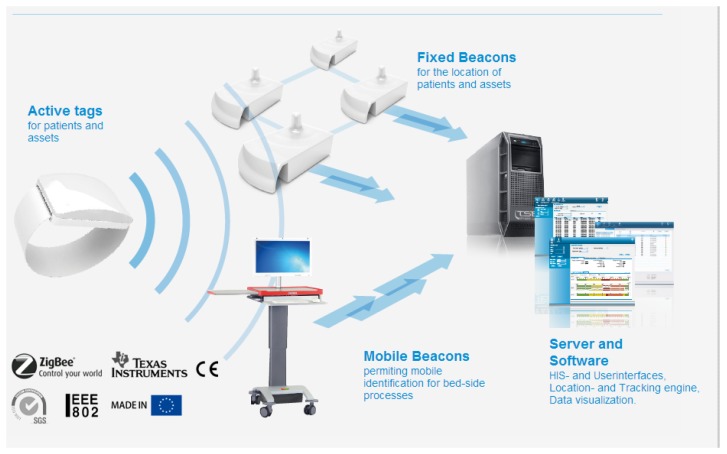
The Sphera System architecture.

In order to collect location data in the environment of the user, an ILS is needed. In the case of the eMotiva Project, the commercial product, Sphera ILS [29], has been selected. Sphera ILS was specifically designed to provide the area location of humans and devices in health centers. The Sphera System is currently installed in La Fe Hospital in Valencia. The Sphera System architecture is presented in Figure 1. This system ensures that the batteries of bracelets can be continuously active for at least one year. Other systems based on other technologies only have sufficient energy to work continuously from one day to one month. This feature is very important, as it is necessary for the user to wear the bracelet for a long time for this experiment. A system involving weekly or monthly battery change for the kind of subjects needed for our study (*i.e.*, elderly people with the risk of dementia) interferes too much with the experiment and heightens the risk of rejection of the bracelet by the user.

The system is made up of a set of beacons that are installed in the areas where the user should be located. The Sphera beacons connect with the Sphera servers using Ethernet protocols. The location is determined using active bracelets that are worn by users. Each bracelet has a unique number, which identifies the user. The bracelets connect periodically to beacons using the Zigbee protocol. All beacons within reach of the bracelet send the bracelet identifier and an estimation of the distance of the beacon to the bracelet to the server at all times. A more in-depth explanation of the Sphera System is out of the scope of this article.

## 4. eMotiva Process Mining Algorithms

In this section, we present the eMotiva process mining algorithms and the results of applying this tool to actual residents of a nursing home involved in the eMotiva Project.

**Figure 2.**
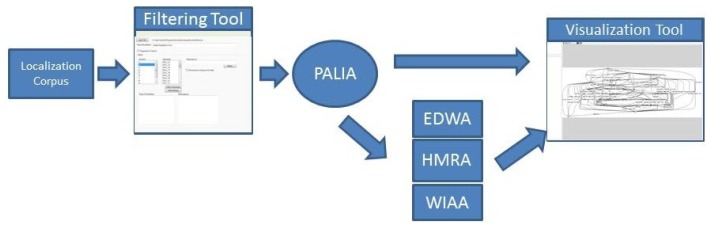
The eMotiva tool.

The eMotiva process mining framework combines algorithms and visualization interfaces in order to infer, process and show workflows from sets of location events. The location events are stored in a log by the Sphera System, which was installed in a nursing home in la Pobla de Vallbona (Spain). The Sphera System adds an entry in the log when a user changes its location area. The events emitted by the Sphera System are stored with information about the identifier for resident, the date and time when the event occurred and the area in which the resident was identified. The eMotiva process mining algorithms use that information to create tracking models for each resident. This tracking information is processed by specific algorithms to highlight the most interesting information in order to help experts. In Figure 2, a general view of the architecture of the eMotiva process mining framework is shown.

The main algorithm in the framework is PALIA (parallel activity-based log inference algorithm) [14]. PALIA is a process mining algorithm that is able to infer workflows from activity log samples. The results of PALIA are timed parallel automatons (TPA) [33]. A TPA is a formal representation framework for workflows that allows for a high expressivity of capabilities whilst maintaining a regular grammar complexity. This ensures the possibility of expressing a very high variety of human behavior patterns using a simple mathematical approach to make the inference more efficient.

The current implementation of PALIA is able to process MxML (Mining eXtensible Markup Language)corpora and to provide files written in DOT language, which is a well known plain text graph description language. This is the standard method that is used in ProM Process Mining tool [36]. ProM is the most widely used application for process mining. This application has a large quantity of different algorithms that can be used to analyze workflow Logs. ProM is a desktop application. We have decided to implement PALIA in an independent way in order to allow it to be accessed by legacy systems as a web service or by automatic tasks to automatically infer and compare the workflows. In order to make it easier for professionals to make inferences of specific moments and individuals, the system provides a filtering module that allows us to create a subcorpus from the whole location corpus. Using that module, it is possible to create tracking models of specific users within specific dates in order to see the model's evolution over time. This filtering module uses the eMotiva algorithms for creating the models and shows them to experts through the graphic visualization module, allowing them to see the inferred workflows in detail. To visualize the workflow, a specialization of the GLEEalgorithm [37] for automatic creation of TPA layouts was implemented. Due to the wide variety of different patterns that can be inferred by PALIA, the size of the resultant models can be very large. As a result, a tool for zooming workflows was created to allow professionals to navigate through the models and see in greater detail any specific part of the model.

Despite the use of PALIA and filtering and visualization tools, finding differences among the inferences at specific moments in time is difficult, due to the size of the models. In order to be able to detect specific problems or behavior changes more easily, specific algorithms have been developed:

- **EDWA (edition distance workflow algorithm):** EDWA is thought to identify a distance measured between two TPAs. For the purposes of our study, that means a measure of the difference between two behavior models. EDWA is designed under the error correcting grammar inference framework (ECGI) [35]. As we are using annotated workflows, its application in this problem is easy. EDWA returns the list of different nodes (for example, physical location) and transitions between two TPAs and a number representing the distance between them. The type of differences that EDWA is able to detect are transitions added or deleted and nodes added or deleted. The distance measured is calculated according to the account of differences detected weighted by their type. It is possible to customize the weights of each type of difference in order to prioritize some differences over others. The general formula is presented in Equation (1):
  (1) $$WD=W_{NA}*\left|N_{A}\right|+W_{ND}*\left|N_{D}\right|+W_{TA}*\left|T_{A}\right|+W_{TD}*\left|T_{D}\right|$$
  where *W_NA_* is the weight for added nodes, *N_A_* is the list of added nodes, *W_ND_* is the weight for deleted nodes, *N_D_* is the list of deleted nodes, *W_TA_* is the weight for added transitions, *T_A_* is the list of added transitions, *W_TD_* is the weight for deleted transitions and *T_D_* is the list of deleted transitions. A formal definition of the algorithm is presented in Algorithm 1.
  EDWA not only provides measurement of the differences between two workflows, but also provides a list of differences that can be highlighted in the eMotiva visualization module in order to allow experts to easily detect the changes existent between both workflows. In our problem, EDWA was used to measure and identify the differences between the behavior models of the residents.
- **WIAA (workflow instance acceptor algorithm):** In addition to comparing two inferred models, sometimes it is necessary to compare a model with a single sample. This comparison allows for the detection of abnormal samples that are not usual in the individual behavior of a resident. WIAA is thought to compare inferred models of behavior of a person with single samples in order to know if the samples are in accordance with the model or not. Where our study is concerned, this algorithm has been used to assist in the identification of undesired outlier samples of residents' behavior. A formal definition of WIAA is presented in Algorithm 2
  WIAA not only calculates if the sample is accepted by the workflow, but also, it is able to provide a list of differences between the sample and the workflow when the sample is not accepted. Using these differences, an edition distance can be implemented, like in EDWA, to provide a measure of the difference between them.

**Algorithm 1** EDWA algorithm.
**Require:** Two TPA to be compared (TPA1, TPA2)**Ensure:** List of different nodes and transitions, (float)Distance Res ← Create List of differences **for all** t ∈TPA1.Transitions **do**  **if** ∄ t in TPA2.Transitions **then**   Res.AddedTransitions.Add(t)  **end if** **end for** **for all** t ∈ TPA2.Transitions **do**  **if** ∄ t in TPA2.Transitions **then**   Res.DeletedTransitions.Add(t)  **end if** **end for** **for all** n ∈ TPA1.Nodes **do**  **if** ∄ n in TPA2.Nodes **then**   Res.AddedNodes.Add(t)  **end if** **end for** **for all** n ∈ TPA2.Nodes **do**  **if** ∄ n in TPA2.Nodes **then**   Res.DeletedNodes.Add(n)  **end if** **end for** **return** Res, CalculateDistance(Res);
- **HMRA (heat maps rendering algorithm)** In addition to comparison algorithms, some characteristics of the models can be highlighted to provide more information about the execution of the process. When the number of transitions occurring between two nodes(representing locations) or the duration of the accumulated execution of determined actions exceed a specific threshold, this could signal the presence of important information that needs to be quickly identifiable by the expert. HMRA calculates the accumulated duration of locations and the number of transitions between them in order to highlight the flows with different colors forming *heat maps*. Heat maps are widely used in ProM plugins to highlight areas in processes for general purposes. Heat maps provide a very useful tool for experts that allows us to automatically highlight the most probable steps and locations in order to detect the most important parts of a workflow on the first viewing. For our study, this algorithm has been used to highlight the favorite locations and behavior patterns of the user.

**Algorithm 2** WIAA algorithm.
**Require:** TPA of the model, List A of actions**Ensure:** List of changes, (Bool) AgTPA? Trans ← Create List of Transitions current ← TPA.InitialState actions ← {} // set of actions active **for all** a ∈ A **do**  actions.add(a)  **if** ∃ t in TPA2.Transitions | t.SourceNodes ∈ actions **then**   Trans.Add (t);   current ← t.ENDSTATE   actions.remove(t.SourceNodes)  **end if** **end for** **if not** Current ∈ TPA.Finals? **then**  Res = CalculateDiferences(TPA,Trans) **end if** **return** Res, Current ∈ TPA.Finals?

## 5. Experimental Results

The eMotiva process mining algorithms were tested with location information collected in a nursing home in La Pobla de Vallbona (Spain). For the experiment, nine patients were chosen to wear a location bracelet for 25 weeks. We chose the most independent patients for this study; old people with the capacity to move freely in the nursing home have been selected. The system gathers information on the location of the patient at any given moment. During this experiment, 125,584 location events have been gathered. For this experiment, the location events were separated by days, so each sample represented a complete day. As a result, the TPAs inferred by PALIA will represent the tracking of a typical day of a resident in a nursing home. The available filtering tool is able to create a training corpus from specific dates. We have divided the experiment into two.

The first experiment is focused on supporting the discovery of sudden changes in individual behavior. In order to do that, it is necessary to detect samples that are different from the usual behavior model of the individual. As the individual behavior model is continuously changing, it is necessary to update the model over time. In this experiment, we infer workflows using one month of samples representing the behavior of the user during this time. We use a month of samples to ensure the variability of behavior depending on the day of the week. Each sample (representing a day) was compared with the model of the previous month in order to detect the differences between the model and daily executions using WIAA. As WIAA returns a list of changes, it can be used to calculate a numeric distance between the model and the sample.

**Figure 3.**
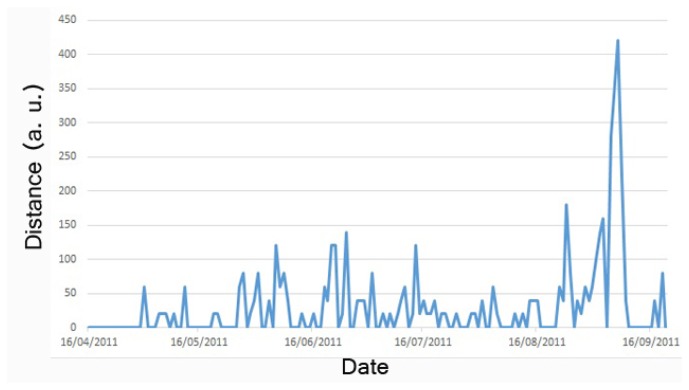
Detection of outliers with the workflow instance acceptor algorithm (WIAA) distance.

Figure 3 presents a curve representing an example of the daily differences for one resident of the nursing home. In the figure, a clear peak can be seen on September 7th that might represent a sudden change in the behavior of the resident.

**Figure 4.**
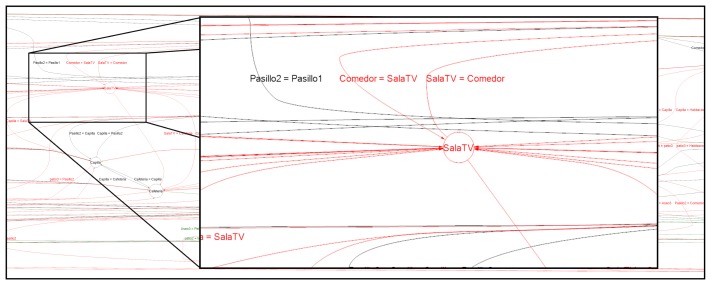
Detail of edition distance between 7th September and the last behavior model learned (August).

**Figure 5.**
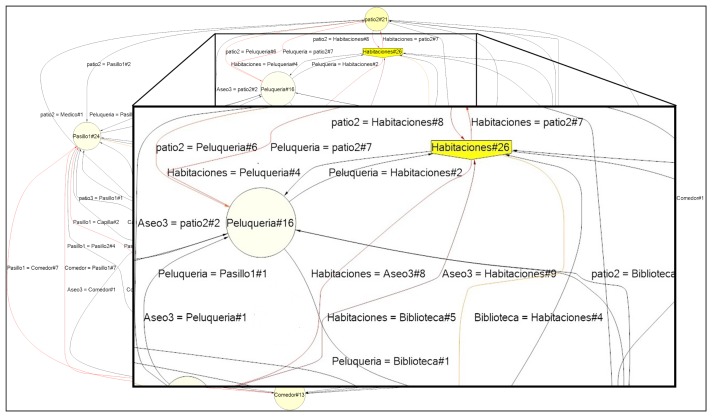
Detail of the heat map of 7th September.

Studying this case in detail, on the one hand, in Figure 4, a comparison between two inferred workflows representing two different weeks is shown. In this figure, it is easy to see that this day, the TV room (SalaTV), is highlighted in red, which means that user had not visited this room, unlike the usual model of the resident. On the other hand, in Figure 5, the heat map of 7th September shows that the user spent a great quantity of time in their personal room (Habitaciones). Using this information and the clinical history, the medical staff is able to detect if this behavior change is due to a temporary illness, to depression or other issues and react accordingly. We do not have access to this information, due to privacy issues.

In the second experiment, we show the behavioral difference over time during the 25 weeks of the data gathering. In this experiment, we calculate a distance between weekly inferred models. We calculate the workflow distance using EDWA and applying correction weights between the differences between the models. Based on previous experiments, we valuate the correction weight of a node as the double of the correction weight of a transition in order to calculate a numerical value for the distance. Using this numerical value, we have calculated two curves. One of the curves represents the weekly behavior change, making a comparison between a week and the next one. The second curve represents the behavior change over time, by comparing the first week with the rest of the weeks. Intuitively, if the behavior model of the individual was static, the slope comparison between the two curves should be similar; however, if the slope of the curves is different, that suggests that there is a dynamic change in the individual behavior of the resident.

**Figure 6.**
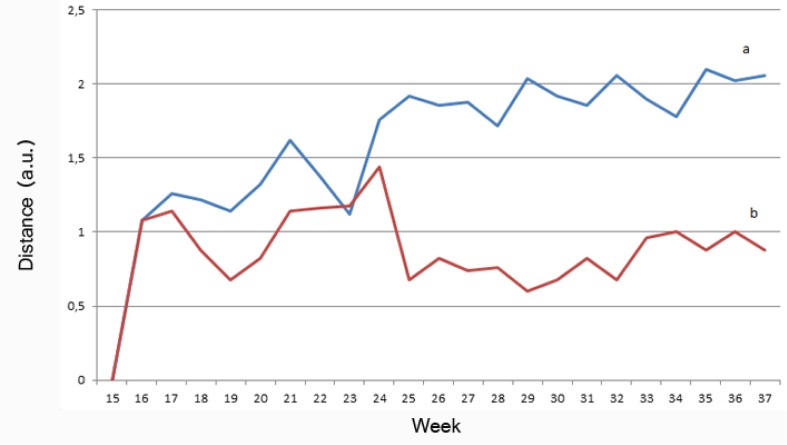
Evolution of workflow distances: (**a**) Absolute workflow distance; (**b**) Partial workflow distance (velocity).

In Figure 6, an evolution of the distance measured over the weeks is shown. In that figure, two lines are represented. Line (a) represents the absolute difference of each inferred model with the workflow of the first week. Line (b) represents the relative difference to the previously inferred workflow. As can be seen, Line (a) tends to slightly grow over time. Line (b) is more or less constant in time. This interesting graph indicates that the behavior of the user is changing in a constant way from one week to another, and the absolute difference of behavior changes is increasing. That suggest that this curve can represent an incremental behavior change (incremental drift) in the individual.

**Figure 7.**
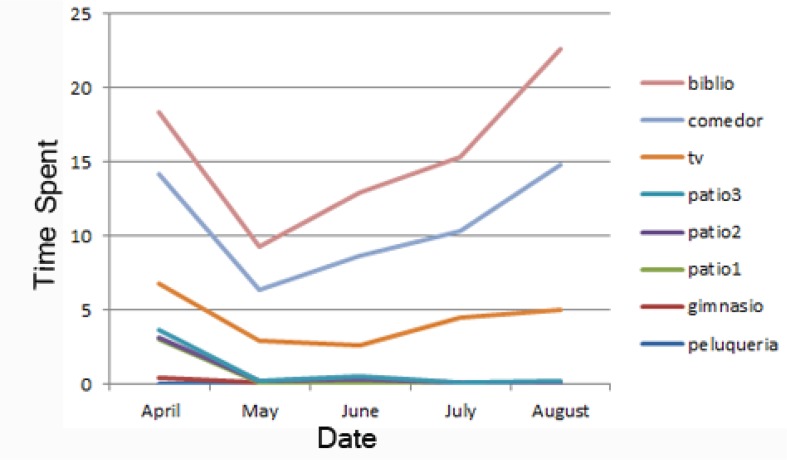
Evolution of favorite areas.

If we look at the amount of time the user stays in a specific area, we can detect how the behavior of the resident changes with respect to their favorite areas. Figure 7 represents the evolution of the length of the stay in the user's favorite areas over the months. For example, in the summer months, the resident spends more time inside the nursing home (TV room (*SALATV*), lunch room (*Comedor*) and library (*biblio)*) than outside (courtyards (*patio 1, 2 and 3*)). This is probably due to the summer heat in Valencia, which can reach more than 40 degree Celsius

## 6. Discussion and Conclusions

To summarize, the tool presented has been used to perform a test of the methodology presented to support experts in human behavior modeling using process mining technology. This methodology has been tested using real patients in a nursing home that have been studied for a 25-week period. In this paper, algorithms that allow for the measurement of the distance between different behavior models based on location have been formalized. These algorithms will enable the quantification of the behavioral change of users. In addition, a tool to apply those algorithms to a Sphera-based corpus is presented. This tool is able to present automatically inferred workflows to experts using heat map algorithms to facilitate its understanding and to highlight the differences in the behavior of users.

We have provided graphical tools to support experts in detecting sudden changes in the behavior of users and the discovery of the reasons for those changes. Furthermore, we calculated a slope that could be used as an indirect measure of the rhythm of incremental behavior change of the user over time. In our opinion, according to this experiment, there are reasons to think that, although the movement of residents in nursing homes is limited, their behavior is continuously evolving and changing. Our life experiences continuously modify our thoughts and our behavioral model. That means that our current behavioral model will probably never be repeated in the future. As a result, the more time we take to build an accurate and specific behavioral model, the less valid it is for the user.

Taking that into account, the use of pattern recognition technologies, which automatically induce models from available samples of the daily actions of users, appears to be the only way to model human behavior and to detect changes in an effective way. Process mining technologies can be used not only to provide these models, but also to permit behavior experts to take a look inside the model in an understandable way. This is because process mining technology sacrifices some inference capability to achieve a greater understanding of the models. In our opinion, this important difference makes the new paradigm of process mining one of the most adequate paradigms to address useful human behavior models.

In any case, the use of process mining technologies is suitable to infer and present individual models to experts that represent human behavior in a visual and understandable way. In addition, the processing of noisy location data with these technologies allows the experts not only to detect the particular behavior patterns of individuals, but also to help detect noisy patterns in order to extract outlier samples from the corpus and to detect ILS system deployment problems.

This study is limited by the number of cases available for observation. For that, in the future, we will enrich the corpus with more samples and over more time. In addition, we plan to enrich the corpus with more information about the user's daily actions in order to test the system with more complex data, thus allowing the creation of more complete behavior models that will empower experts in such a way that the detection of individual patterns and their changes will be possible.
